# Life Cycles of Myxogastria *Stemonitopsis typhina* and *Stemonitis fusca* on Agar Culture

**DOI:** 10.1111/jeu.12754

**Published:** 2019-09-12

**Authors:** Dan Dai, Benjamin A. Okorley, Yu Li, Bo Zhang

**Affiliations:** ^1^ College of Plant Protection Shenyang Agricultural University Shenyang Liaoning 110866 China; ^2^ Engineering Research Center of Chinese Ministry of Education for Edible and Medicinal Fungi Jilin Agricultural University Changchun Jilin 130118 China

**Keywords:** Aphanoplasmodia, morphogenesis, ontogeny, spore‐to‐spore culture, Stemonitales

## Abstract

Myxogastria is a group of protozoa characterized by cellular uninucleate amoeboflagellates (myxamoebae and flagellated swarm cell), acellular multinucleate plasmodia, and stationary spore‐bearing sporocarps. The Stemonitales is a large order in the Myxogastria and contains approximately 230 species, but only 13 species have their completed life cycles observed so far. Here, we described the life cycles of two species in Stemonitales, *Stemonitopsis typhina* and *Stemonitis fusca* by culturing in water agar medium and observing the morphogenesis of their spore germination, plasmodium, and sporocarp development. The spore‐to‐spore life cycles of *Ste. typhina* and *S. fusca* were completed in approximately 67 and 12 d, respectively. Both species possessed an aphanoplasmodium. However, the spores of *Ste. typhina* and *S. fusca* germinated by the V‐shape split and pore methods, respectively. Unlike *S. fusca* with an evanescent peridium, *Ste. typhina* produced a shiny persistent peridium which was continuous with the membrane surrounding its stalk. The information will contribute to a better understanding of their taxonomy and phylogeny.

THE true slime molds (myxomycetes or Myxogastria) is the largest group in the phylum Amoebozoa (Adl et al. 2012; Kang et al. 2017), which is a group of protozoa characterized by cellular uninucleate amoeboflagellates (myxamoebae and flagellated swarm cell), acellular multinucleate plasmodia, and stationary spore‐bearing sporocarps.

The sporocarp of true slime molds possesses structural characteristics that are important and form the basis of the current taxonomic system in the Myxogastria (Clark and Haskins 2014). Therefore, knowledge on the character development and variations in sporocarp morphology is critical to proper recognition of species and the construction of a realistic taxonomy and systemetics. In the Myxogastria, normal little variations occur among specimen of the same species. However, irregular phenotype forms can be produced by abnormal or unstable environmental conditions during sporulation. This has made the discovery of artificial methods of inducing myxogastria sporulation very important especially in studying their development from spore‐to‐spore (Alexopoulos 1969).

Until 1960, the taxonomy of the Myxogastria was almost entirely based on the morphology of the fruiting body structures (sporocarps, hypothallus, peridium, stalk, columella, capillitium, and spores). Information about the life cycle characteristics was rarely mentioned in many descriptions of species. Studies on the living phases of their life cycle such as the amoeboflagellate, the plasmodium, and sporocarp development unraveled other developmental characteristics that were lost in the sporocarp forms. Martin (1960) proposed the new order, Echinosteliales, based on in vitro experimental results in support of taxonomic conclusions above the species level. Later, Ross (1973) built a new subclass, the Stemonitomycetidae, based on two types of sporocarp development: suprahypothallic and subhypothallic.

*Stemonitopsis* is a subgenus of the genus *Comatricha* which was later raised to the generic level based on having a partial surface net and a stalk composed of more or less intertwining fibers at least at the base (Nannenga‐Bremekamp 1991). It is a genus between *Stemonitis* and *Comatricha* that looks more similar to *Stemonitis* for its cylindrical sporotheca and partial surface net. *Stemonitopsis typhina* (F. H. Wigg.) Nann.‐Bremek. has a worldwide distribution and occurs on soft decaying wood (Takahashi 2004). Among the ten species of *Stemonitopsis* (Kirk et al. 2008; Lado 2005–2019), *Ste. typhina* is the only species with a stalk surrounded by a membrane (Nannenga‐Bremekamp 1991). The capillitium formation of *Ste. typhina* has been studied by Ross (1957) based on materials collected from the field, but its complete life cycle and how the membrane is formed have not yet been described.

*Stemonitis*, on the other hand, is a common genus of Stemonitidaceae. The spore‐to‐spore life cycle of five (*Stemonitis flavogenita* E. Jahn, *S. fusca* Roth, *S. herbatica* Peck, *S. splendens* Rostaf., and *S. virginiensis* Rex) out of its 19 species have been completed in vitro (Kirk et al. 2008; Lado 2005–2019). However, for *S. fusca*, the most widely distributed myxogastria all over the world, detailed micrographs showing the series of events occurring during the development of morphological characters are lacking (McManus 1961; McManus and Richmond 1961).

In this study, the complete spore‐to‐spore life cycles of *Ste. typhina* and *S. fusca* were induced in water agar media to provide detailed information about their external morphology and events that characterized their life cycles.

## Materials and Methods

### Field sampling

The sporocarps of *Ste. typhina* and *S. fusca* used in the study were obtained in moist chamber culture of substrates collected from Hongsongwang scenic spot (127°86′17″–127°89′04″E, 42°45′33″–42°45′96″N, on July 6, 2016) and the campus of Jilin Agricultural University (125°24′22″E, 42°48′33″N, on July 16, 2017), respectively. The substrates (decayed wood) were air‐dried and transported to the laboratory in sealed plastic bags. Moist chamber cultures were set up in the manner described by Dai et al. (2018). All specimens used in the experiments were stored in herbarium boxes along with detailed information (dates of germination, plasmodium formation, and fruiting and also the medium and the name of the experimenter). The specimens were deposited in the herbarium of the Mycology, Engineering Research Center of the Chinese Ministry of Education for Edible and Medicinal Fungi, Jilin Agricultural University, China (HMJAU‐M1556, HMJAU‐M1557).

### Identification

Observation and measurement of sporocarp morphological characteristics were carried out with a LEICA M165 continuously variable microscope (Leica Microsystems, Wetzlar, Germany) and a Zeiss Axio Imager A2 microscope (Carl Zeiss Microscopy LLC, Jena, Germany). Stained slide mounts were prepared according to the method described by Zhang and Li (2013). Ten air‐dried sporocarps and 20 spores mounted in 3% KOH were observed and measured under an oil immersion objective lens (100X). Sporocarps were also prepared for scanning electron microscopy (SEM) as described by Zhang and Li (2016). SEM analyses and photomicrographs of specimens were carried out using a SU8010 scanning electron microscope (Hitachi, Tokyo, Japan). Briefly, several sporocarps were attached to a holder, coated with gold using a Hitachi E‐1010 sputter and then examined.

### Germination (suspension culture)

Three sporocarps each from *Ste. typhina* (obtained 4 d after sporulation) and *S. fusca* (obtained 87 d after sporulation) from the first generation were crushed to release spores in a 1.5 ml centrifuge tube containing 200 μl sterilized distilled water. The tubes were maintained at a constant temperature (25 °C) and observed periodically (every 10 min for *Ste. typhina;* every 30 min for *S. fusca*) in order to record the time of spore germination. Observation slides were prepared according to Dai et al. (2017). A LEICA DM2000 microscope (Leica Microsystems), Zeiss Imager A2 microscope (Carl Zeiss Microscopy LLC), and a Zeiss 710 Laser scanning confocal microscope (Carl Zeiss Microscopy LLC) were used to obtain photomicrographs of amoeboflagellates (myxamoebae and flagellated swarm cells).

### Agar culture

After suspending the spores of *Ste. typhina* (after one day) and *S. fusca* (2 d), the spore suspension of each species was added to 3 ml of sterilized distilled water and then was poured into a 2% water agar medium to observe their plasmodial formation. A little oat powder and decayed tree log powder were added to the medium contained in the 9 cm Petri plates. The plates were observed every two days and the time for plasmodial formation recorded. The mature plasmodia were then sub‐cultured in a 2% water agar medium to keep the plasmodia alive.

## Results

### *Stemonitopsis typhina* life cycle and morphology in agar culture

#### Spore germination

**Figure 1:**
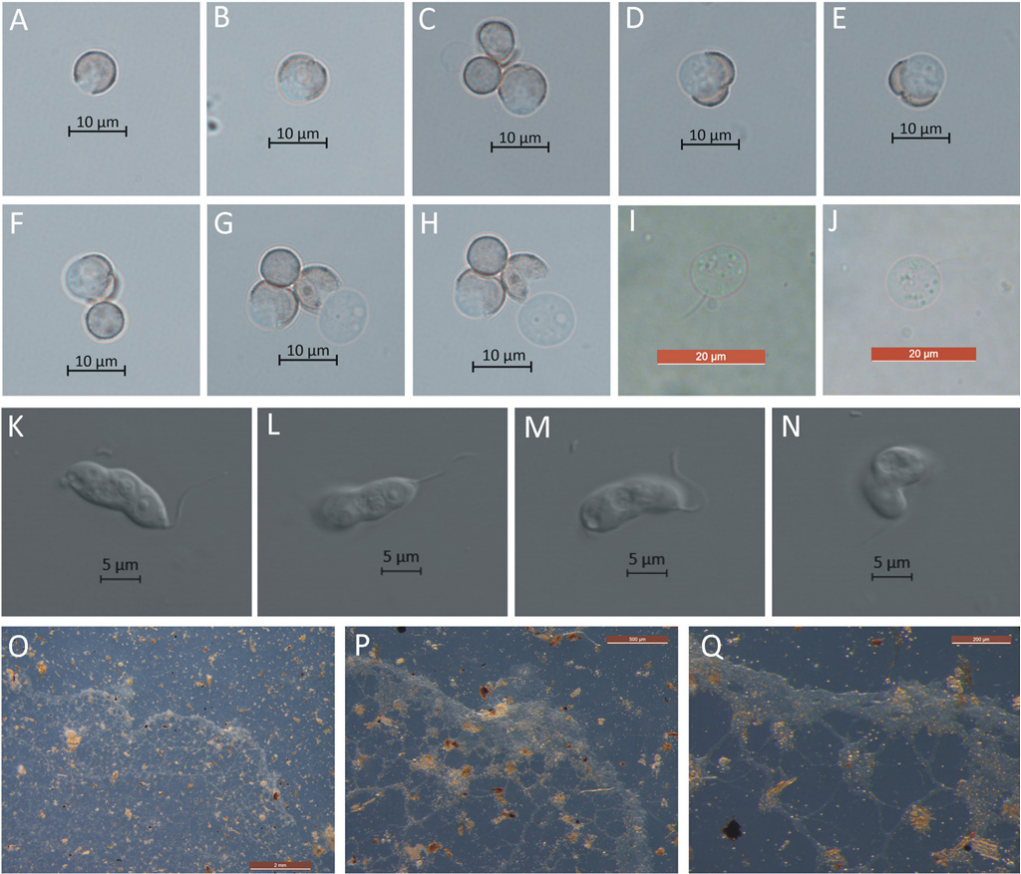
Spore germination and plasmodium of *Stimonitopsis typhina*. **A–H.** Germinating spores with a V‐shape split in the spore wall 10 min postsuspending spores. **I, J.** Swarm cell 10 min postsuspending spores under a light microscope. **K–N.** Swarm cell two days postsuspending spores under a laser scanning confocal microscope. **O.** Aphanoplasmodium on water agar medium 64 d postsuspending spores. **P, Q.** Leading edge of aphanoplasmodium. Scale bars: A–H = 10 μm; I, J = 20 μm; K–N = 500 μm; O = 2 mm; P = 500 μm; Q = 200 μm.

Ten minutes after suspending spores in sterile water, the spores began to germinate and release one protoplast by the V‐shaped split method (Fig. 1A–H). The protoplast was transparent, round, 7.5–10 μm in diameter. This then changed into a short cylindrical swarm cell with a single flagellum attached at the tapering end (Fig. 1I, J). The swarm cells were transparent and bigger than the protoplast two days after spore germination (Fig. 1K–N).

#### Plasmodium

Sixty‐four days after spore germination, the mature plasmodia of *Ste. typhina* were observed on the 2% water agar surface. The plasmodia were transparent, flat and with thin veins on the surface of the medium (Fig. 1O), typical of aphanoplasmodia. It also had a fan‐like advancing margin that exhibited polarity (phaneroplasmodium) (Fig. 1P) and showed reversible protoplasmic streaming under a dissection microscope (Fig. 1Q). Preservation of the mature trophic stage of the plasmodium was successful for 2 yr by sub‐culturing it every month in fresh 2% water agar media sprinkled with sterile oak and decayed tree log powder.

#### Sporulation

Sporulation was observed when Petri plates containing the crawling phaneroplasmodia were exposed to alternating diffused light and dark photoperiods (about 15 h of light and 9 h of darkness, for approximately 2–6 photoperiods). The first indication of fruiting was the emergence of anomalous milky‐white protuberances over the surface of the 2% agar medium.

**Figure 2:**
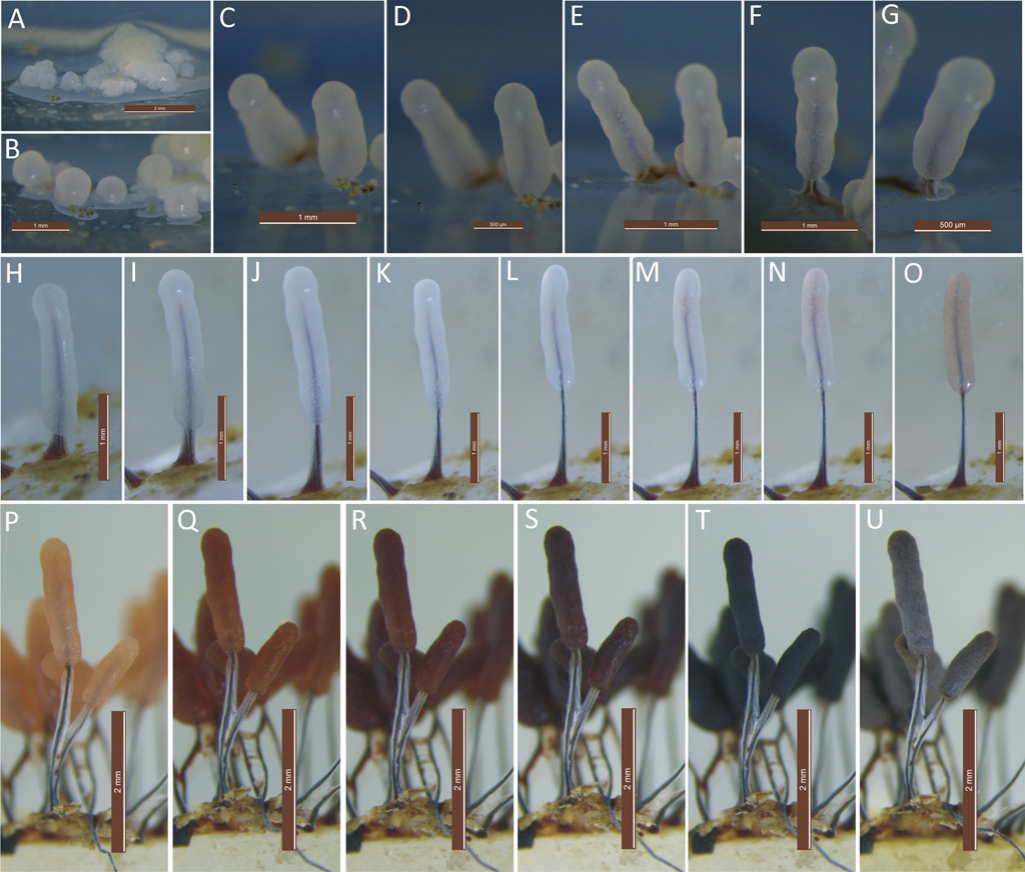
Sporulation of *Stemonitopsis typhina*. **A–E.** Period of sporotheca formation. E, **F–L.** Period of stalk formation. L, **M–U.** Period of sporocarp maturity. Scale bars: A = 2 mm; B, C = 1 mm; D = 500 μm; E, F = 1 mm; G = 500 μm; H–O = 1 mm; P–U = 2 mm.

The sporulation of *Ste. typhina* can be divided into three periods: (i) Sporotheca formation (Fig. 2A–E). During this period, the anomalous protuberances gradually became hemispherical and then spherical (Fig. 2A, B), leading to the formation of the young sporotheca. The young sporotheca changed from spherical to a cylindrical shape, developed to a size similar to its mature sporotheca (0.8–2.1 mm), ahead of stalk formation (Fig. 2C–E). (ii) Stalk formation (Fig. 2F–L). After the developing sporotheca attained its full height (1.0–2.1 mm), the stalk of *Ste. typhina* formed by the simultaneous elongation at the top of the cylindrical sporotheca and constriction at the base along the inner columella until the stalk reached its maximum height (2.3–3.8 mm). The process left behind a membranous material surrounding the entire stipe. The period lasted about 3 h (at 20 °C). (iii) Sporocarp maturity (Fig. 2M–U). This period was marked by changes in sporotheca color from white to pinkish, to pink, to red‐brown, and finally to a dark gray color as the spores developed. The maturation process lasted about 4 h, while the entire sporulation of *Ste. typhina* took about 19 h to compete.

**Figure 3:**
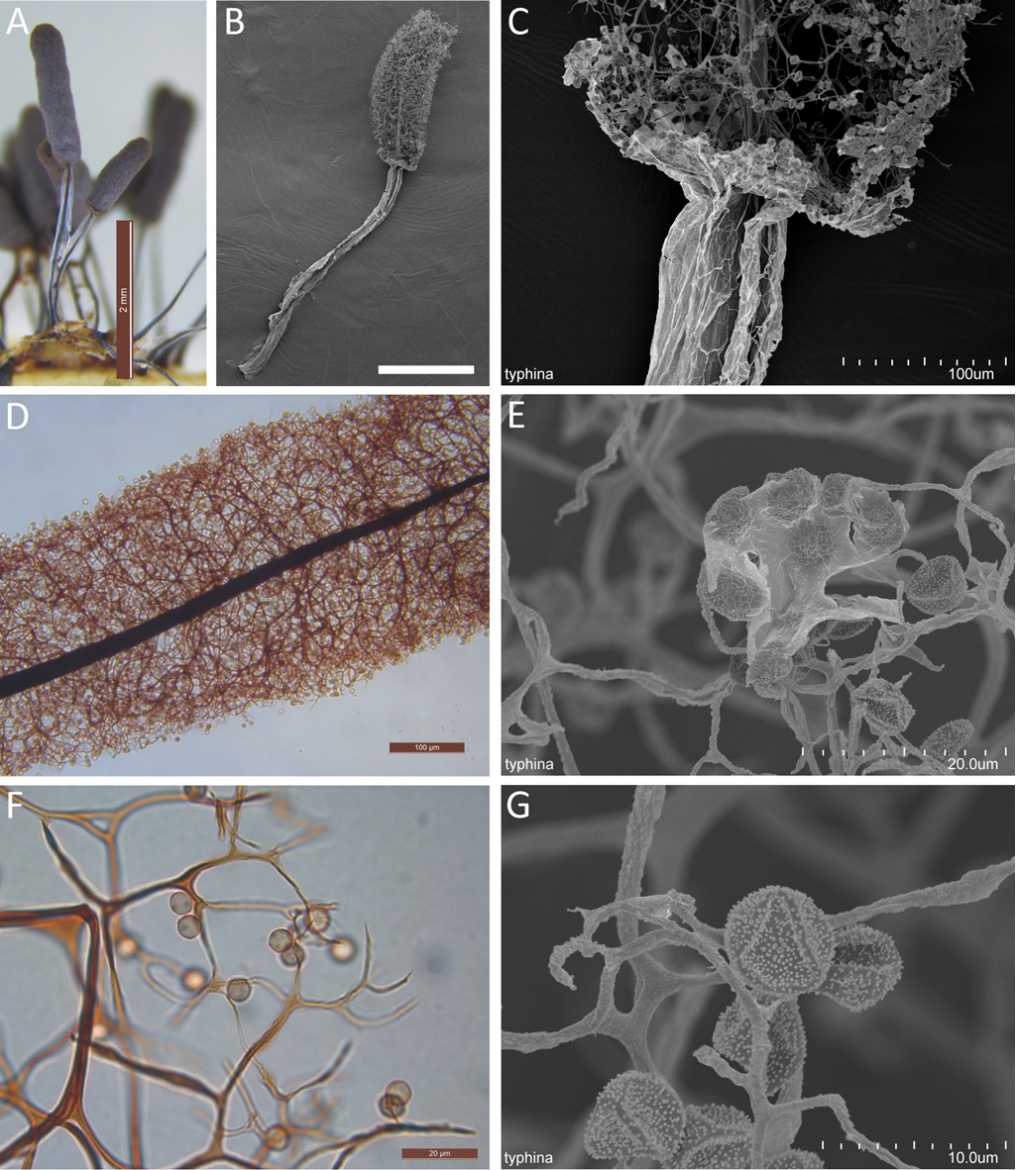
*Stemonitopsis typhina*. **A.** Sporocarps grew on an agar medium. **B.** Sporocarps by SEM. **C.** The base of sporotheca shows the continuous of peridium with the membrane surrounding its stipe. **D.** Part of sporotheca by transmitted light. **E.** Peridial fragment in the surface of sporotheca covers several spores by SEM. **F.** Spores by transmitted light. **G.** Spore by SEM. Scale bars: A = 2 mm; B = 1 mm; C, D = 100 μm; E, F = 20 μm; G = 10 μm.

By slowly decreasing the humidity of the culture, the sporocarps began to dry, and the peridium eventually became lilac‐gray (Fig. 2U). The peridium of *Ste. typhina* was continuous with the membrane surrounding the stalk (Fig. 3A–C). The spore‐to‐spore culture of *Ste. typhina* was repeated twice, each time yielding the same results. In addition, the spores of the first generation were still viable.

The species description of *Ste. typhina* below is based upon sporocarps collected from the moist chamber and water agar cultures (Fig. 3A–G). The sporocarps were scattered to gregarious, stipitate, cylindric, obtuse, erect, lilac‐gray with a silvery shine that was lost to reveal brown, 2.3–3.8 mm total height, and 0.3–0.4 mm in diameter. The stalks were black and shiny and surrounded by a silvery membrane up to half the total height and merging into the peridium. The hypothallus was membranous and red‐brown. The peridium was lilac‐gray and shiny silvery in reflected light, dehiscing into large flakes and often disappearing completely except for occasional persistence as irregular patches or basal collars or cups. The capillitium was densely reticulate, arising from the entire columella, branching and anastomosing pale brown filaments that ended freely with no surface net. The columella was tapering, reaching nearly the apex of the sporotheca. The spores were brown in mass and lilac‐brown in transmitted light, marked with clusters of larger warts scattered on the wall surface (Fig. 3F, G), and 6.0–7.0 μm in diameter. The height of sporocarps (2.3–3.8 mm), sporotheca (0.8–2.1 mm), stalk (1.0–2.1 mm), and the diameter of spores (6.0–7.0 μm) in agar culture sporocarps were bigger than that of the height of sporocarps (3.0–3.8 mm), sporotheca (1.5–2.1 mm), stalk (1.5–2.0 mm), and the diameter of spores (6.5–7.0 μm) in moist chamber cultured sporocarps.

### *Stemonitis fusca* life cycle and morphology in agar culture

#### Spore germination

**Figure 4:**
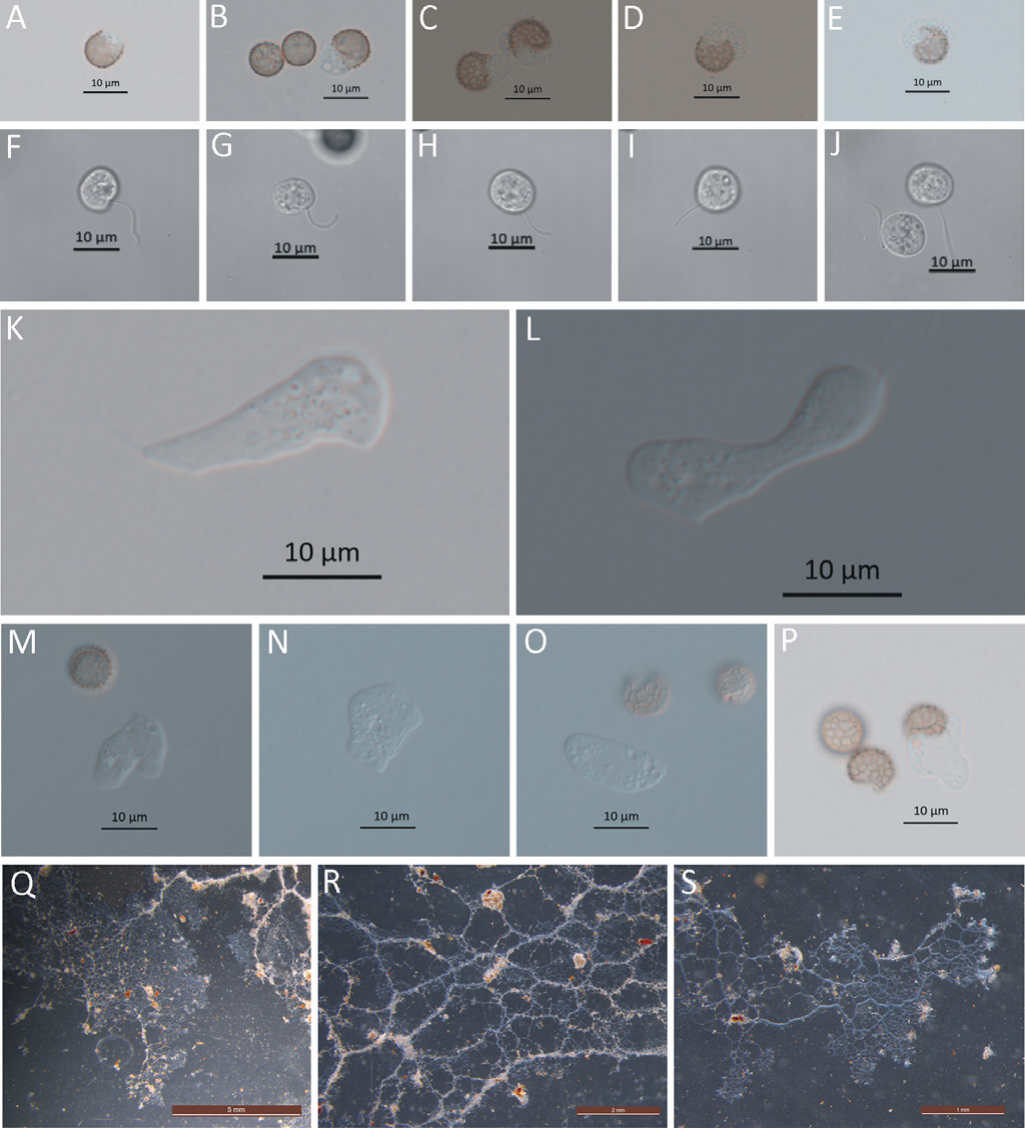
Spore germination and plasmodium of *Stimonitis fusca*. **A–E.** Germinating spores with a pore in the spore wall 90 min postsuspending spores. **F–J.** Swarm cell with one obvious flagellum under a laser scanning confocal microscope. **K, L.** Swarm cell on the surface of agar medium under a light microscope. **M–P.** Myxamoebae. **Q–S.** Aphanoplasmodium on the surface of petri dish. Scale bars: A–P = 10 μm; Q = 5 mm; R = 2 mm; S = 1 mm.

About 90 min after suspending spores in sterile water, a rounded pore (about 4–6 μm) appeared at the spore wall as the protoplast oozed outward (Fig. 4A–E). The protoplasts were transparent and about 7.5–10 μm in diameter. In the following 30 min, the protoplast changed into a spherical swarm cell with one clear flagellum on the observation slide (Fig. 4F–J). However, when humidity is decreased on the water agar surface, the swarm cells gradually became irregularly shaped with one clear flagellum (Fig. 4K, L) and took on the appearance of myxamoebae (Fig. 4M–P).

#### Plasmodium

About 8 d after spore germination, the mature plasmodia were observed on the surface of the 2% agar medium containing a little pine and oat powder, and a covering layer of water (Fig. 4Q–S). The plasmodia were transparent, thin and with advancing edges that showed reversible protoplasmic streaming under a dissection microscope. The veins were watery white and conspicuous on the surface of the Petri plate glass (Fig. 4Q). The coralloid plasmodia grew and moved for 2–3 h to reach the edges of the Petri dish. Compared to *Ste. typhina*, the plasmodial growth of *S. fusca* was abundant and rapid in the culture.

#### Sporulation

**Figure 5:**
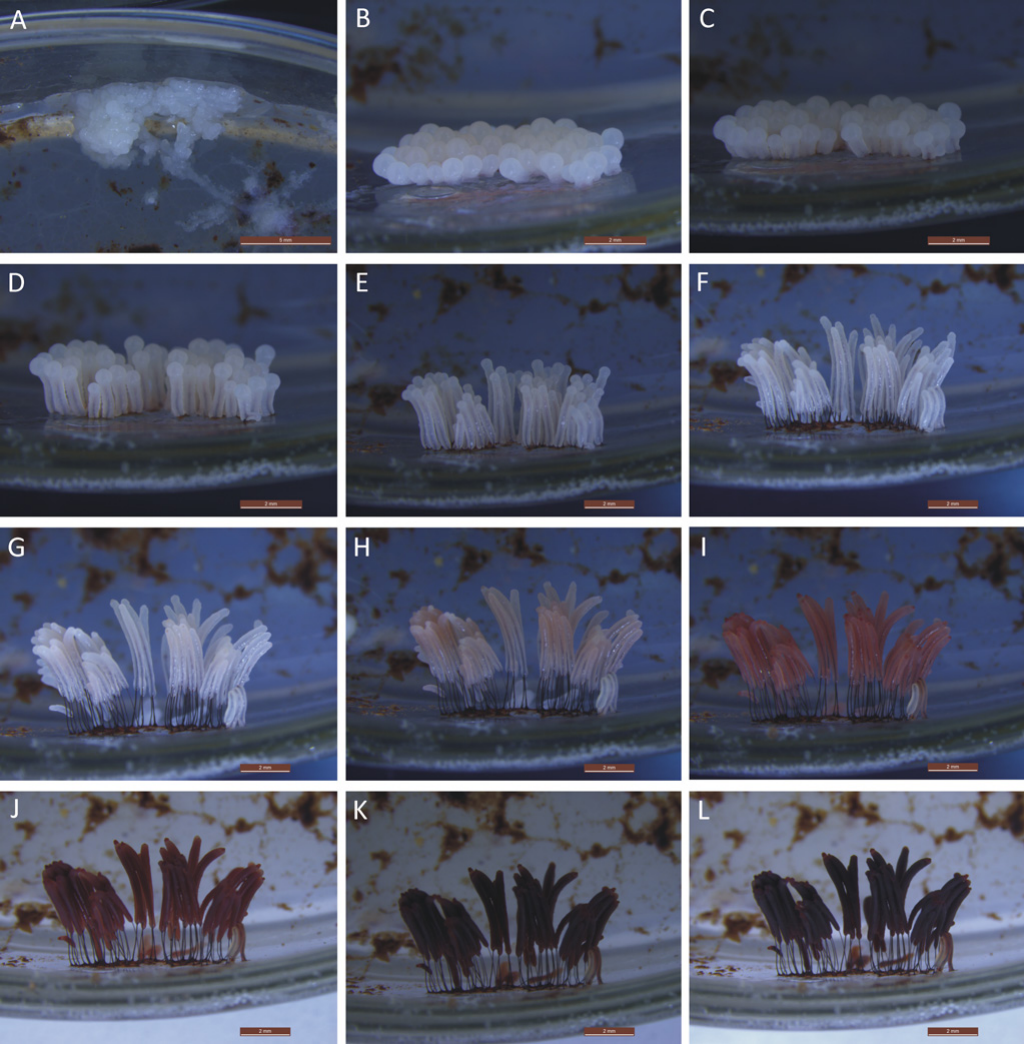
Sporulation of *Stemonitis fusca*. **A–E.** Period of sporotheca formation. **E**,** F, G.** Period of stalk formation. **G**,** H–L.** Period of sporocarp maturity. Scale bars: A = 5 mm; B–L = 2 mm.

Sporulation began with the appearance of milk‐white coralloid plasmodia after exposure to natural sunlight (12 h of light and 12 h of darkness for three photoperiods in sunny weather). The sporulation of *S. fusca* can be divided into three periods: (i) Sporotheca formation (Fig. 5A–F). During this period, the coralloid plasmodium formed an anomalous mass and then separated into several primordia (Fig. 5A, B). The hypothallus was laid first underneath the primordium by protoplasmic secretion before primordial elongation. Gradually, the individual primordia elongated as the columella formed inside (the young sporotheca), and finally assumed the cylindrical shape of a mature sporotheca (Fig. 5C–F). This process lasted for 133 min. (ii) Stalk formation (Fig. 5F, G). When the sporotheca had attained full height (5.5–7.2 mm), the stalk, already formed as columella inside the sporotheca started to show as the sporotheca moved upwards. The sporotheca remained white until the full stalk height was reached (1.7–2.5 mm). This period took 36 min (at approximately 23 °C). (iii) Sporocarp maturity (Fig. 5H–L). In the following 150 min, when the stalk had attained full height, the color of the sporotheca changed from white to pinkish, to pink, to reddish‐brown, and finally became dark as the spores developed. The entire sporulation process took about 11 h to complete. The shiny immature peridium that is present in the early stages of sporocarp maturity eventually disappeared. The spore‐to‐spore culture of *S. fusca* was repeated twice, each time yielding the same results.

**Figure 6:**
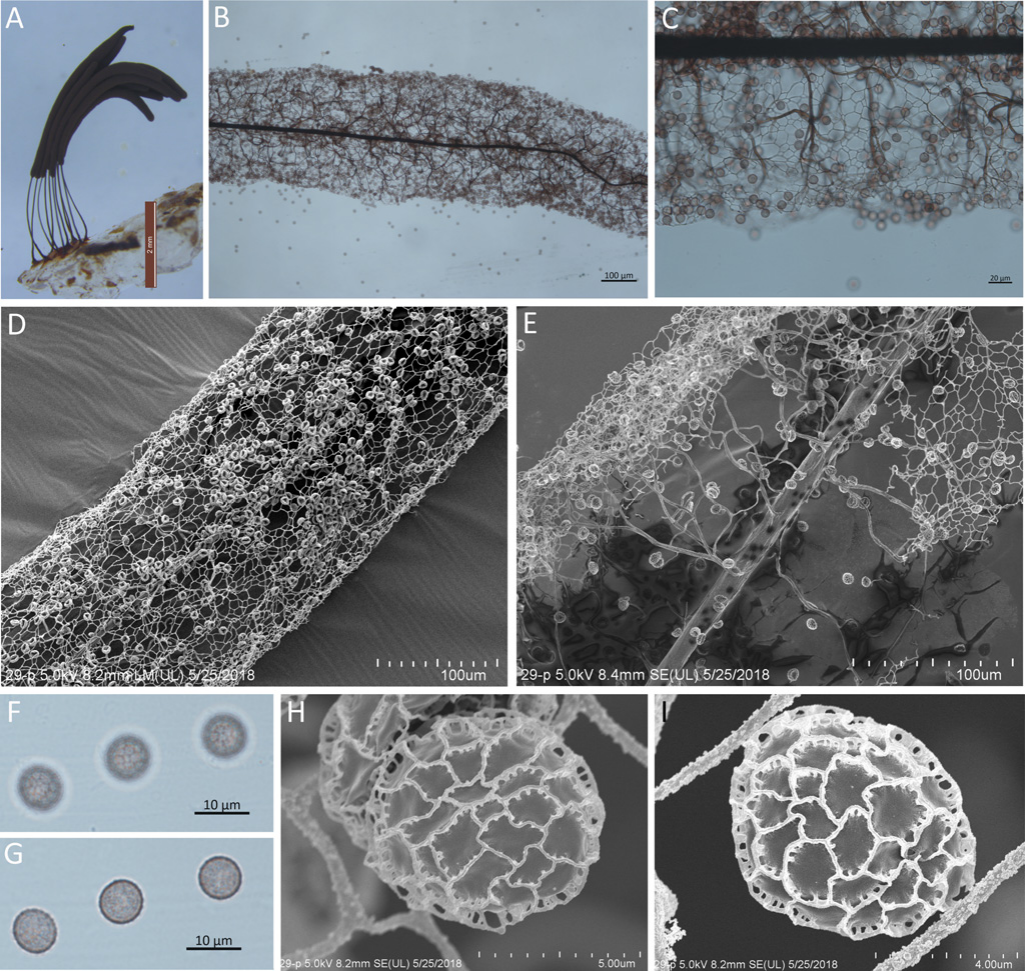
*Stemonitis fusca*. **A.** Sporocarps grew on an agar medium. **B.** Part of sporotheca by transmitted light. **C.** Part of the surface net by transmitted light. **D.** Part of sporotheca shows surface net by SEM. **E.** Part of sporotheca shows capillitium by SEM. **F, G.** Spores by transmitted light. **H, I.** Spore by SEM. Scale bars: A = 2 mm; B, D, E = 100 μm; C = 20 μm; F, G = 10 μm; H = 5 μm; I = 4 μm.

The species description of *S. fusca* below is based on sporocarps collected from the water agar culture (Fig. 6A–I). Sporocarps were gregarious, more or less cylindrical, erect, stood on a common hypothallus, entire height with stalk about 5.5–7.2 mm. Peridium fugacious. Stalk black, polished, 1.7–2.5 mm long. Columella central, running almost to apex of sporotheca, then subdivided into several branches. Capillitium, violet‐black in the center, arcuate, flexuous, forming a loosely meshed central reticulation which becomes dark violet as it nears the surface. Surface network complete being usually irregular or falling away in the apex. When perfect, the meshes of the surface net vary from 12 to 20 μm in diam. Spores, spinulose‐reticulated (Fig. 6H, I), 7–8.5 μm in diam., nearly black in mass, deep blackish violet singly under the microscope.

## Discussion

This study document the morphogenesis of *Ste. typhina* and *S. fusca* in water agar culture medium. Spores of *Ste. typhina* germinated by the V‐shaped split method after 10 min of incubation to release a single protoplast and the entire life cycle was completed in approximately 67 d. Spores of *S. fusca* germinated by the pore method after 90 min of incubation but completed its entire life cycle in a short time (about 12 d) relative to *Ste. typhina*. Although factors including pH, temperature, and spore age affect spore germination rate in slime molds (Gray and Alexopoulos 1968; Smart 1937), the spores of both *Ste. typhina* and *S. fusca* germinated easily at 25 °C with no adjuvants included to the sterilized distilled water.

Spore internal pressure and enzyme activity are known to induce the V‐shape split and minute pore germination method, respectively (Gilbert 1928). In this study, the spore germination method was influenced by interspecific differences. Like *Collaria arcyrionema* (Rostaf.) Nann.‐Bremek. ex Lado (Dai et al. 2018), *Ste. typhina* is the second species in Stemonitales to employ the V‐shaped split method for spore germination under a similar cultural condition. The pore method of spore germination in *S. fusca* agree with those observed in *Amaurochaete comata* G. Lister & Brândza, *Comatricha nodulifera* Wollman & Alexop., *C. orthotricha* Bratteng, *Stemonitis herbatica* Peck, *S. splendens* Rostaf., and *S. virginiensis* Rex (Alexopoulos 1959; Bratteng 1975; Dai et al. 2017; Farr 1982; Indira 1969; Mims 1973; Wollman and Alexopoulos 1968; Yang 1968). The presence of the two methods of spore germination in the same order agrees with the recognition of Stemonitales as a paraphyletic group in the Myxogastria (Fiore‐Donno et al. 2008).

Three major plasmodial morphotypes have been described in the Myxogastria—phaneroplasmodium, aphanoplasmodium, and protoplasmodium (Alexopoulos et al. 1996). Of the species of myxogastria with their life cycle completed, all 13 species belonging to Stemonitales reported till date possessed an aphanoplasmodium (Chen et al. 2013; Clark 1995; Dai et al. 2018; Gao et al. 2017; Kalyanasundaram 1974; Lado et al. 2007; Li et al. 2017; Liu et al. 2010; Song et al. 2014; Wrigley de Basanta et al. 2008, 2009, 2011, 2012; Zhu et al. 2019). Similarly, the plasmodial characteristics of both *Ste. typhina* and *S. fusca* were consistent to the aphanoplasmodium, which favors Alexopolous's opinion that the morphology of the plasmodium might be a useful taxonomic criterion for species delimitation (Alexopoulos 1960).

The prefructification coralloid plasmodium was not observed in *Ste. typhina*. In *S. fusca,* a white and opaque coralloid plasmodium was observed in the present study, which corroborates that observed by McManus (1961). The formation of coralloid plasmodium in the other three species of *Stemonitis* has also been described and serves as the signal for the beginning of fructification (Dai et al. 2017; Indira 1971). These descriptions included the movement of the coralloid plasmodium in *S. herbatica* (Dai et al. 2017; Indira 1971), the short distance movement of the coalesced plasmodium in *S. virginiensis* (Mims 1973; Mims and Rogers 1973), and the formation of a thick and milky‐white coralloid mass in *S. flavogenita* (Alexopoulos 1959). Except for *S. splendens* which lacks the coralloid stage, the prefructification coralloid stage is perhaps only found in forms like *Stemonitis* with a clustered fruiting body. However, this need to be confirmed by studying the life cycles of other *Stemonitis* species.

Further, the nonpigmented aphanoplasmodia of both *Ste. typhina* and *S. fusca* failed to sporulate when incubated in darkness, except after exposure to diffused light. Similar studies on sporulation of nonpigmented plasmodia were reported to occur either in light or in darkness, while yellow pigmented plasmodia always required light for sporulation (Gao et al. 2017; Keller and Schoknecht 1989). However, the results of the present study suggested that light had a clear effect on inducing sporangia formation or on development during the life cycles of *Ste. typhina* and *S. fusca*.

The peridium is essential but a neglected character in the taxonomy of Myxogastria (Fiore‐Donno et al. 2008). Although the sporotheca shape of *Ste. typhina* and *S. fusca* were similar, the peridium of *S. fusca* showed up in the early stages during sporulation and finally disappeared. While in *Ste. typhina*, it persisted as a shiny peridium. The presence of the persistent peridium of *Ste. typhina* confirms phylogenetic evidence that *Ste. typhina* is closely related to *Comatricha nigra* (Pers. ex J.F. Gmel.) J. Schröt., which also has a persistent peridium (Zhang et al. 2018).

Gray (1936), noted that the stipe of *Ste. typhina* formed after the immature cylindrical sporotheca reached the maximum height of the sporocarp in a study on stipe formation. However, a different result was obtained in the current study. The stipe of *Ste. typhina* developed simultaneously as the immature cylindrical sporotheca elongated at the top and constricted at the base along the inner columella, leaving behind a membrane surrounding the entire stipe (Fig. 2H–L). As a result, the peridium of *Ste. typhina* was continuous with the stipe membrane, making it the major difference between its variety *Ste. typhina* var. *similis* (G. Lister) Nann.‐Bremek. & Y. Yamam. It was reported in Stemonitales that the stalk is internally secreted by the cytoplasm (Indira 1971; Mims 1973; Ross 1973; Yang 1968), while in Physarales, it is merely a constriction of the cytoplasm and its surrounding membrane (Haskins et al. 1978). Therefore, *Ste. typhina* is typified by stalk formation patterns of both Stemonitales and Physarales.

The stipe of *S. fusca* developed without the constriction of the immature sporotheca, and there was no membrane surrounding the stipe. A tentative inference on this result is that a stipe with or without a surrounding membrane can be used as a stable morphological feature representing two types of stipe formation in Stemonitales. However, there are very few studies where the sporocarp development has been reported in detail and further work is still needed to complete as many as possible the life cycles of the remaining species in Stemonitales.

In conclusion, we investigated the detailed spore‐to‐spore life cycles of *Ste. typhina* and *S. fusca*, especially the external morphological development of the sporocarp to supplement information for taxonomic decision and phylogeny. Further studies might focus on the internal morphology during sporulation, especially on the capillitial, partial surface net, and spore formation.
